# Progress in Surgery

**Published:** 1898-02-05

**Authors:** 


### Progress in Surgery.

### SURGERY OF THE LIVER AND GALL-BLADDER.

**Movable Liver.**—A case is reported by Keen[1] where a floating liver and distended gall-bladder was mistaken for a floating kidney in a woman, aged 44. There were several gall-stones. Cholecystotomy was performed, and the gall-bladder fixed to abdominal wall, so affording anchorage for the liver. The pains from which the patient suffered were cured. Packard[2] also reports a case of movable liver, but before operation was performed the patient died of cerebral hæmorrhage. Peters operated on a case for supposed hydronephrosis, and found it to be one of movable liver. On the other hand, a carcinoma of the mesentery has been mistaken for a movable liver. Winkler attributes the displacement to pendulous abdomen and stretching of the suspensory ligament. Faure maintains that the true suspensory ligament of the liver is the coronary ligament, or rather the numerous cellular and vascular bands contained between its layers, and the chief support furnished by the inferior vena cava.

**Penetrating Wounds of Liver** are summarised by Kerr and Ford.[3] Total number of cases, 51; recovered, 33; died, 18; mortality, 35·2 per cent. Edler deduces the general mortality of liver wounds in 543 cases (no treatment specified) as 66·8 per cent.; and Mayer gives the mortality of 61 gunshot wounds as 34·4 per cent. The author prefers suturing to tamponnage, as the mortality is lower, and they favour an elastic suture, as recommended by Babacci, for the following reasons: (1) It is perfectly supported by the animal tissues and the part of the liver which encapsulates it; (2) it can be threaded in a needle smaller than its own diameter by cutting it obliquely; (3) it enters the wound in the hepatic capsule with facility (if a round needle be employed), because its elasticity renders it slender when it is drawn through the tissues, and when traction stops it thickens and perfectly fills up the hole through which it has passed; (4) unlike other threads, it does not rub or saw in its passage; it lacerates less and passes more easily, and is less liable to cut its wayout; and (5) in the liver, an organ subject to great normal oscillations in volume, the elastic suture causes less tension than does any other, for it yields before congestion, yet holds the surfaces apposed when the organ shrinks. Elastic sutures should never be fastened with knots, but the ends should be doubled over upon themselves about half a centimetre and tied with silk or fine catgut, while held close to the edge of the tissue. Terrier and Auvray[4] are in favour of immediate laparotomy in cases of wound of the liver or bile-ducts. They are thus opposed to Routier, who holds that, in cases of rupture of the bile-ducts, the operation is more successful when performed after the acute symptoms have subsided. Moore[5] and Martin[6] both report cases of rupture of the liver successfully treated by laparotomy. The latter ascribes the result to the free irrigation of the peritoneal cavity with hot water, by which the bleeding was checked, and to the thorough drainage.

**Resection of the Liver** for multilocular hydatid cyst is reported by Bruns.[7] The cyst, which was about the size of a man's fist, was embedded in the hepatic tissue. The gall-bladder, which was adherent to the tumour, was first dissected out, and then the cyst was excised by cutting freely into the substance of the liver. Only two arteries had to be tied. The edges of the hepatic wound were sutured deeply with strong catgut, and also more superficially with fine catgut. This case is the first in which resection of the liver has been performed for a multilocular cyst, and the abdominal cavity immediately closed after excision of the tumour. The case was successful.

**Diagnosis of Neoplasms of Liver** can often not be made without exploratory operation, and even then not without puncture, which, as Tuffier[8] says, has too often proved fatal from the profuse and irrepressible hæmorrhage which has resulted. In all cases of doubt the hilum of the liver should be explored; if enlarged glands are present, they show a metastasis from a malignant growth, and puncture is unnecessary; but their absence does not exclude a secondary malignant growth, because then the metastasis would not be through the lymphatics, but through the venous channels, and the glands would not be enlarged. He proposes, as a means of hæmostasis, the temporary digital compression of the pedicle of the liver, the left index finger being introduced through the foramen of Winslow, and compression made by the thumb of all the structures of the pedicle.

**Hepatic Abscess** is characterised, acccording to Johnston,[9] by history in which dysentery and chill appear, general malaise, pain and tendency over the liver, hectic, sweats and rigors, right-side posture, erratic temperature, progressive emaciation, and gastric disturbance. Incision, either directly or through the pleura after resecting one or more ribs, is the only safe procedure. Berger[10] narrates a case of abscess which occurred six years, and Render[11] another which occurred ten or twelve years, after suffering from dysentery. O'Conor[12] reports several interesting cases of hepatic abscess operated upon by the trans-thoracic route.

**Cholelithiasis.**—Beck[13] points out the great value of Carlsbad water in this condition. It induces peristalsis and stirs up the circulation in the abdominal organs, particularly the liver, where so often cholangeitis and cholecystitis are present as a consequence of cholelithiasis. Such inflammatory processes are often cured; at least the stone becomes quiescent, and the cholelithiasis becomes, as Riedel says, "latent." He advises operation in the following conditions: (1) Whenever the diagnosis of acute cholecystitis is made, cholecystotomy should be performed without delay; (2) cholecystotomy should also be performed in chronic hydrops of the gall-bladder; (3) whenever acute colicky attacks in the region of the gall-bladder, combined with fever, return for a second or third time; (4) whenever jaundice is present for more than four weeks; (5) in gall-stone ileus; and (6) in all obscure cases where inflammatory symptoms resembling peritonitis occur in the neighbourhood of the gall-bladder. Robson[14] gives the following statistics: Of 170 operations of all classes, including malignant cases, 10 died. Of the 93 patients without jaundice all recovered, all the deaths occurring amongst the 77 jaundiced cases. Out of 115 cholecystotomies there were 5 deaths, 3 being the subjects of cancer and 2 of suppurative cholangitis with jaundice; in other words, the mortality of cholecystotomy for gall-stones is only a little over 1 per cent. There were 7 cholecystectomies with 1 death, 14·25 per cent. Of 26 cholelithotrities all recovered. Of 6 cases of choledochotomy 1 died, 16·6 per cent. Of 11 cholecystenterostomies 1 died, and that patient was the subject of extensive malignant disease. Of 11 cases operated upon for separation of adhesions, 3 for intestinal obstruction, and 1 for peritonitis, all recovered. He would operate for the following conditions: (1) In frequently recurring biliary colic without jaundice, with or without enlargement of the gall-bladder; (2) in enlargement of the gall-bladder without jaundice, even if unaccompanied by great pain; (3) in persistent jaundice ushered in by pain, and where recurring pains, with or without ague-like paroxysms, render it probable that the cause is gall-stones in the common duct; (4) in empyæma of the gall-bladder; (5) in peritonitis, starting in the right hypochondrium; (6) in abscess around the gall-bladder or bile-ducts, whether in the liver, or under, or over it; (7) in some cases where, although gall-stones may have passed, adhesions remain, and prove a source of pain and illness; (8) in fistula, mucous, muco-purulent, or biliary; and (9) in certain cases of chronic jaundice, with distended gall-bladder dependent on some obstruction of the common duct, although the suspicion of malignancy be entertained. In such cases the increased risk must be borne in mind, as malignant disease may be the cause of the obstruction, and operation in such cases is attended with greater danger than ordinary; (10) in phlegmonous cholecystitis and in gangrene, if the case be recognised at a sufficiently early stage of the disease; (11) in gun-shot injury or in stab-wounds over the region of the gall-bladder; (12) in suspected rupture of the gall-bladder without external wound; (13) in some cases of chronic catarrh of the gall-bladder or bile-ducts; and (14) in suppurative cholangitis. Homans[15] gives an interesting case in which large gall-stones formed around silk sutures 20 months after recovery from cholecystotomy, and Stirling[16] a case in which he removed 175 gall-stones from a woman, aged 42. Robson endeavours to avert the danger of hæmorrhage in deeply-jaundiced patients by the administration of 30-grain doses of chloride of calcium every four hours for a few days before the operation, as recommended by Dr. Wright.[17]

[1] Med. Chron., June, 1897, p. 189. [2] Ibidem. [3] Med. News, Aug. 14, 1897, p. 203. [4] Med. Week, July 9, 1897. [5] Lancet, Sept. 18, 1897, p. 722. [6] Birming. Med. Rev., May, 1897, p. 291. [7] Med. Week, July 9, 1897. [8] Amer. Jour, Med. Sci., Ap., 1897, p. 483. [9] Med. Rec. New York, June 5, 1897, p. 822. [10] Med. Week, July 16, 1897. [11] Ibidem. [12] Glasgow Med. Journ., May, 1897, p. 339, and Annals of Surg., May, 1897. [13] New York Med. Jour., May 8, 1897, p. 631. [14] Lancet, June 19, 1897, p. 1,666. [15] Annals of Surgery, July, 1897, p. 114. [16] Intercolon. Med. Journ. of Australas., July 20, 1897, p. 465. [17] *See* Brit. Med. Jour., Dec. 19, 1891.

